# A Cross-Sectional Study of Labial Bone and Covering Soft Tissue in Maxillary Anterior Segment: A Dilemma in Orthodontics

**DOI:** 10.1155/2021/5553301

**Published:** 2021-07-12

**Authors:** Saeed Yousefzadeh, Maryam Johari, Sedigheh Sheikhzadeh, Sina Haghanifar, Hemmat Gholinia, Nazanin Arbabzadegan Hashemi

**Affiliations:** ^1^Student Research Committee, College of Dentistry, Babol University of Medical Sciences, Babol, Iran; ^2^Department of Oral and Maxillofacial Radiology, Oral Health Research Center, Health Research Institute, Babol University of Medical Sciences, Babol, Iran; ^3^Department of Orthodontics, Oral Health Research Center, Health Research Institute, Babol University of Medical Sciences, Babol, Iran; ^4^Oral Health Research Center, Health Research Institute, Babol University of Medical Sciences, Babol, Iran; ^5^Health Research Institute, Babol University of Medical Sciences, Babol, Iran; ^6^Oral Maxillofacial Radiologist, Babol University of Medical Sciences, Babol, Iran

## Abstract

**Purposes:**

The thickness of the buccal bone and its covering gingiva is pivotal in determining the prognosis of implant therapy as well as fixed orthodontic appliances, especially nonextraction treatments. The purpose of this study was to evaluate the buccal bone thickness and covering soft tissue in the maxillary anterior segment.

**Methods:**

This study measured the hard tissue thickness at 2 and 5 mm more apical from the crest and at the root apical apex, as well as the distance from the CEJ to the alveolar crest, using 80 CBCT images divided into three age groups. In addition, the distance from free gingiva to alveolar crest and from free gingiva to CEJ was measured. The acquired data then was analyzed using an ANOVA, *t*-test, and Pearson correlation to investigate any associations or statistically significant differences between parameters.

**Results:**

The highest mean soft tissue thickness at the 5 mm level was for central incisors and the least for canine. The highest mean thickness of soft tissue at the crest level and its 2 mm apical level was related to central incisors and the lowest mean thickness at these levels was related to canine. Analysis of hard tissue variables showed the lower thickness of hard tissue at higher ages compared to the young patients group, but the thickness of the soft tissue increases with age.

**Conclusion:**

The highest mean thickness of the buccal hard tissue in the maxillary anterior segment was in lateral and central incisors. Also, the most prominent thickness of the labial soft tissue was in the central and lateral incisors at levels close to the crest.

## 1. Introduction

The maxillary anterior segment plays an important role in having a better smile and thus enhancing the patient's self-confidence. In order to obtain a favorable outcome in cosmetic dentistry such as orthodontic and implant treatments, special attention must be paid to the morphological features of periodontal tissue such as buccal bone and covering soft tissue thickness [1]. A thick biotype provides a higher aesthetic of the implant and better covers the prosthetic components of the implant [1, 2] and also plays an important role in the outcome of periodontal treatments such as root covering procedures [3–5]. In the treatment of implants with fresh socket technique, the width of the facial alveolar bone is an important factor for long-term success [6]. The initial thickness of the maxillary bone plays an important role in determining the final level of hard and soft tissues following a tooth extraction, as well as selecting the appropriate technique (immediate/early/delayed) for implantation, and reducing subsequent complications [7].

Because bone resorption occurs in the direction of tooth movement, the reduced volume of the alveolar bone is sometimes associated with minimal thickness. Biological and biomechanical factors determine potential side effects of orthodontic treatment such as external root resorption, dehiscence, fenestration, and gingival recession [8]. Many factors may be involved in gingival recession, which are effective in the development or progression of gingivitis and the formation of dehiscence [9]. Potentially, gingival recession is often associated with alveolar bone resorption. Alveolar dehiscence is a defect that results in the crestal bone margin being lowered to the exposed root level. The fenestration is a separate region where the root passes through the bone and the root surface is covered only by the periosteum and gum [10].

To avoid the occurrence of dehiscence and fenestration, knowing the alveolar morphology before orthodontic treatment is of particular importance [11]. Therefore, the evaluation of periodontal biotype is also important in orthodontic treatments. Buccal surface bone thickness and covering gum are also pivotal in fixed orthodontic treatments, especially nonextraction treatments leading to upper incisor protrusion [12]. Moreover, miniscrew placement in the maxillary anterior segment for the correction of problems such as excessive overbite requires knowledge of the thickness of soft tissue and bone in this segment.

There are also various methods for measuring soft tissue thickness such as injection needle, transgingival probing, histology sections, cephalometric radiography, transplantation probes, ultrasonic instruments, and cone-beam computed tomography (CBCT) [13]. The CBCT is a convenient technique for dental and soft tissue anatomy [4] and has the advantages of high diagnostic value and accurate measurement of periodontal width [14]. Although numerous researches have been done on measuring the thickness of buccal bone and soft tissue, only a limited number of studies [4, 15] have evaluated the relationship between these two parameters. Therefore, this cross-sectional study was performed to measure hard tissue thickness and its covering soft tissue in the maxillary anterior segment by CBCT, and to analyze the correlation between hard and soft tissue thickness in this segment.

## 2. Materials and Methods

In this cross-sectional study, the buccal bone and soft tissue thickness in the maxillary anterior segment was examined in 80 CBCT images of the patients aged 16 to 67 years (40 males and 40 females) who were referred to the Oral and Maxillofacial Radiology Center for various reasons such as bone examination for implant treatment. For estimating the required sample size for tests at a significance level of *α* = 0.05, a power of 90%, and compensation for 20% subject withdrawal, a minimum of 68 subjects were to be included. All patients participating in this study were informed about the use of their data in the study, and their names were also excluded from radiography. All CBCT radiographs were prepared using Planmeca (Finland, Helsinki) with 80 *∗* 110 mm FOV (Field of View), voxel resolution of 150 *μ*m, and Romexis software.

To avoid any bias, the patients were selected using a set of inclusion criteria: (a) the presence of all 6 maxillary anterior teeth, (b) the absence of severe rotation or crowding, normal inclination of upper incisors, extensive subgingival restorations, pathological lesion, extensive bone resorption, and fracture in maxillary anterior teeth, (c) and no history of previous orthodontic and periodontal treatments, both clinically and radiographically.

**Figure 1:**
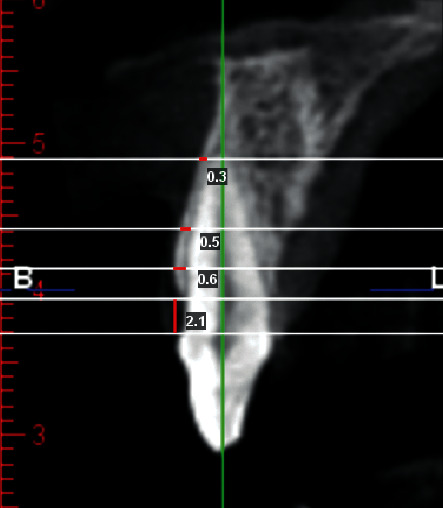
Sagittal section of central incisors (measurement of the thickness of buccal cortical hard tissue in 2 and 5 mm from crest, root apex, and CEJ—C distance).

The hard tissue thickness was measured in sagittal plan, in three different areas (1) at a distance of 2 mm more apical from the crest, (2) 5 mm more apical from the crest, and (3) at the root apical apex perpendicular to the internal cortical bone at each tooth position. In addition, the distance from the CEJ to the alveolar crest was also measured (Figure 1).

**Figure 2:**
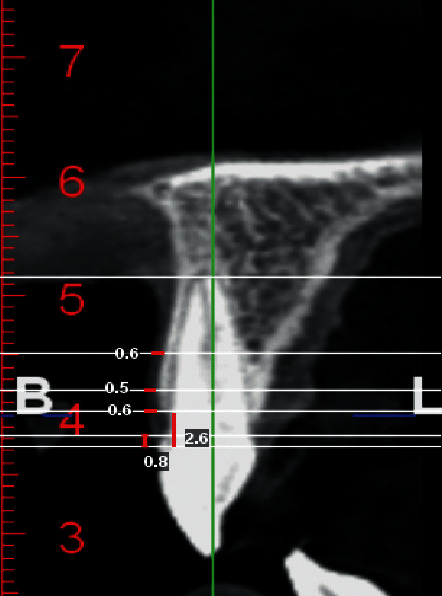
Sagittal section of lateral incisors (measurement of the thickness of buccal soft tissue at crest levels, 2 and 5 mm from the crest, and (FG—CEJ, FG—C distance).

**Figure 3:**
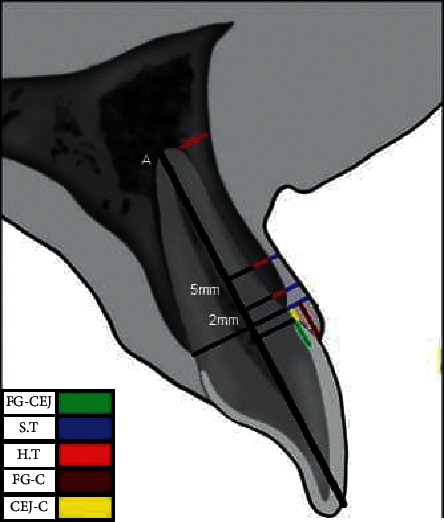
Schematic view of measurements taken for each tooth.

For measuring the thickness of soft tissue, (1) the gingival thickness at the crest level, (2) 2 and 5 mm more apical from the gingiva to the alveolar crest, and (3) from the free gingiva to the CEJ were measured (Figure 2).  Figure 3 represents the schematic view of measurements taken for each tooth.

Additionally, in order to assess the impact of age on bone loss and the thickness of soft tissue, the patients were investigated in three age groups of less than 30 years, between 30 and 40 years, and more than 40 years. The required data was imported into a checklist through radiographic observation of patients. Data were then inserted into Statistical Package for the Social Sciences (SPSS) software v.21 in order to perform the statistical tests. The statistical tests include ANOVA (Analysis of Variance) and Student's *t*-test to understand if there is a significant difference in hard and soft tissue thicknesses between different groups.

To analyze the potential correlations between hard and soft tissue thicknesses, based on tooth and quadrant, Pearson correlation has been utilized to determine if there is any linear association between these two values. Since in ANOVA test it is assumed that the sample values follow the normal distribution, D'Agostino-Pearson has been applied to avoid any bias or wrong assumptions. The test results, based on the calculated skewness and kurtosis, confirmed the normality of the sample data on hard and soft tissue thicknesses (*P* value = 0.037).

## 3. Results

The highest mean thickness in the 2 mm level was related to lateral incisors and the least related to canine, which was not statistically significant. The highest mean thickness at the 5 mm level was related to central incisors and the lowest was related to canine, which was statistically significant (*P* value = 0.002). The highest mean thickness in hard tissue (apical) was related to lateral incisors and the lowest mean thickness was related to central incisors, which was not statistically significant.

In the examination of soft tissue variables according to Tables 1–3, the highest mean thickness in soft tissue crest level and soft tissue level 2 mm from crest was related to central incisors and the lowest mean thickness at these levels was related to canine, which was not statistically significant. The highest mean thickness at the soft tissue level (5 mm) was related to central incisors and the lowest mean thickness was canine, as in previous levels, which was statistically significant (*P* value = 0.001). In examining the distance between the crest and the CEJ (CEJ—crest), the highest mean was related to canine and the lowest mean was seen in central incisors, which was statistically significant (*P* value = 0.007).

In the variables related to the height of the free gingiva (FG) from the CEJ and the free gingiva from the crest bone (FG-C), the highest mean FG-CEJ was for lateral incisors and the lowest mean was in relation to canine, which was statistically significant (*P* value = 0.007). The highest mean FG-C was for lateral incisors and the lowest mean was related to central incisors, which was not statistically significant (Table 4).

According to comparing all variables between the left and the right quadrants (Table 1), the mean hard tissue thickness at the left H.T (5) and H.T (A) levels was slightly higher, which was not statistically significant. The soft tissue thickness was only slightly higher at the ST (C) level on the left than on the right, which was not statistically significant. All the measured distances including CEJ-C, FG-C, and FG-CEJ were slightly more on the left which were not statistically significant (Table 1). There was no statistically significant difference in the parameters measured between men and women in the study population according to Table 2 except for HT (5), which was slightly higher in men.

In comparing the age groups in the variable HT (2), the highest mean thickness was related to the age group less than 30 years, and the lowest mean thickness was related to the age group of between 30 and 40 years, which was not statistically significant. Concerning the HT (5) (*P* value = 0.01) and HT (A) (*P* value ≤ 0.001), the highest mean thickness was in the age group of less than 30 years, and the lowest mean thickness was in the age group more than 40 years, which was statistically significant. In the soft tissue thickness at the crest level (*P* value = 0.021) and the soft tissue thickness at the 2 mm level from the crest (*P* value ≤ 0.001), the highest mean was in the age group more than 30 years, and the lowest mean was in the age group less than 30 years, which was statistically significant. The highest mean thickness of ST (5) was in the age group of 30 to 40 years and the lowest mean was in the age group less than or equal to 30 years, which was not statistically significant. Regarding the FG-C and CEJ-C variables, the highest mean was related to the age group more than 40 years and the lowest mean was related to the age group less than 30 years, which was statistically significant (*P* value = 0.009) (Table 3).

On the correlation between hard and soft tissue thickness in right and left quadrant anterior teeth, the highest correlation was related to canine and at the 5 mm level from the alveolar crest, which was statistically significant (*P* value = 0.025 on the right, *P* value = 0.002 on the left). At the 2 mm level, the highest correlation was related to central incisors, which was statistically significant (*P* value ≤ 0.001 on the right, *P* value = 0.015 on the left) (Table 5).

## 4. Discussion

The present study was conducted to determine the correlation of hard and soft buccal tissue thickness in the maxillary anterior segment using CBCT.

The results of this study showed that the lowest hard tissue thickness was observed in the canine teeth at the 5 mm level of the alveolar crest and the highest hard tissue thickness was observed in the lateral incisors at the apical level. In the study of Gakonyo et al., the highest mean hard tissue thickness was in the central incisor at the midlevel root, and the lowest mean hard tissue thickness was in the midlevel root of the canine [16]. The difference in results could be due to the fact that they examined the bone thickness only at two levels of 4 mm from the CEJ and the midlevel root. According to a study by Farahmand et al. [17], the lowest hard tissue thickness was in the lateral incisors at the 8 mm level of the alveolar crest, and the highest thickness was in the canine at the 2 mm level from the crest. The reason for this difference could be the difference in hard tissue thickness points (in the present study, measurements were made at the apical level of root rather than at the 8 mm level of the alveolar crest). In a study by Esfahanizadeh et al., the highest hard tissue thickness was in the lateral incisors at the 5 mm level and the lowest thickness in the canine at the 5 mm level of the alveolar crest, which was close to the results of the present study [7].

In the present study, the highest soft tissue thickness was seen in the central incisors at the alveolar crest level, and the lowest soft tissue thickness was in the canine at the 2 mm level from the alveolar crest. In a study by Kim et al., the highest soft tissue thickness was at the alveolar crest level of the central incisor, and the lowest thickness was at the lateral incisors and the 1 mm level of the alveolar crest [18]. Their study was similar to the results of the present study in terms of maximum soft tissue thickness. For the lowest soft tissue thickness, the difference may be due to race or sample size (20 samples versus 80 samples in our study). In the study by Esfahanizadeh et al. [7], the highest mean soft tissue thickness was in the central incisor and at the alveolar crest level, and the lowest soft tissue thickness was in the canine and at the 2 mm level of the alveolar crest. The analysis of the results showed that there was a similarity in the highest and the lowest soft tissue thickness between their study and the present study, but the lowest soft tissue thickness in the canine was measured at the 2 mm level by Esfahanizadeh et al. [7], and in the alveolar crest level in the present study. This difference is minor and may be due to differences in the statistical population.

In the present study, the maximum distance from CEJ to alveolar crest was related to the canine, and the least value of this parameter was to the central incisor. In the study by Farahmand et al. [17], the maximum distance from CEJ to alveolar crest was related to the canine, and the lowest mean was to the central incisor. In a study of El Nahass and Naiem [19], who examined only the central and lateral incisors, the minimum mean distance from CEJ to crest was found in females and maximal mean distance in males, both of which were observed in the central incisor. In the study of Esfahanizadeh et al. [7], as in the present study, the maximum CEJ to crest distance was related to the canine and the lowest to the lateral incisor. In the studies by Zekry et al. [6] and Januário et al. [20], the lowest mean distance from CEJ to alveolar crest was in the central incisor and the maximum of this parameter was related to the canine, which was consistent with the present study.

In the present study, the highest buccal hard tissue thickness was observed in males and at the apical level, and the lowest buccal hard tissue thickness was seen in females at the 5 mm level of the alveolar crest. Also, the highest buccal soft tissue thickness was in females and at the alveolar crest level, and the lowest buccal soft tissue thickness in males and at the 2 mm level. In the study by AlTarawneh et al. [21], the highest buccal hard tissue thickness was in the men and middle third of the root and the lowest buccal hard tissue thickness was in females and the apical third of the root. In the study of El Nahass and Naiem [19], the lowest hard tissue thickness was in females at a 4 mm level from the crest, which is close to the result of the present study. The only difference is in the lowest buccal hard tissue thickness in females, which was at the 4 mm level in their study and the 5 mm level in the present study, probably due to differences in the research methodology.

In the present study, the highest hard tissue thickness was observed in the age group less than or equal to 30 years in the apical segment, and the lowest hard tissue thickness in the age group of 30 to 40 years at the 5 mm level. In the study by Januário et al. [20], the highest hard tissue thickness was reported in the age group less than 20 years, and the lowest hard tissue thickness was reported in the age group more than 60 years, which was approximately consistent with the results of the present study. In the study of Zekry et al. [6], the highest thickness was related to the age group of less than 30 years, and the lowest mean thickness was related to the age group of more than 50 years. As can be seen, the majority of results in this area show a trend of decreasing hard tissue thickness with age.

In the present study, in the evaluation of correlation between hard and soft tissue thickness in the right and left quadrant anterior teeth, the highest correlation was observed in the central incisor and at the 2 mm level from the crest. At the 5 mm level, the highest correlation was related to the canine. In the study by Esfahanizadeh et al. [7], the highest correlation was observed between hard and soft buccal tissue thickness at the central incisor and then in the canine, which is consistent with the present study. Kheur et al. [22] examined central incisors and reported a correlation between hard and soft buccal tissue thicknesses of 0.49, which was also statistically significant, confirming the results of the present study.

## 5. Conclusion

To avoid the occurrence of dehiscence and fenestration, knowing the alveolar morphology, including the thickness of hard and soft tissue in the maxillary anterior segment, before orthodontic treatment is of particular importance. Therefore, this cross-sectional study was performed not only to measure these two parameters using CBCT but also to analyze the correlation between them considering their location. The results of this study indicated that the highest mean thickness of the buccal hard tissue in maxillary anterior segment was in lateral and central incisors and often in root apical levels. In addition, the most prominent thickness of the labial soft tissue was in the central and lateral incisors at levels close to the crest (0–2 mm to the crest), and caution should be exercised in orthodontic and implant treatments in the canine site. In addition, there was no difference in all variables between the left and right quadrants. In examining the gender variable, only a slight difference was observed in the hard tissue, which was slightly higher in males. The highest correlation was observed in the thickness of soft and hard tissue in the central incisor (the 2 mm level from the crest) and the canine (the 5 mm level from the crest). In analyzing soft and hard tissue variables at different ages, it was found that the thickness of soft and hard tissue at older ages was generally lower than that of young individuals.

## Figures and Tables

**Table 1:** Comparison of hard and soft tissue thicknesses in maxillary anterior segment measured based on left and right quadrants.

Quadrants	Right	Left	Significance level
	Mean ± SD	Mean ± SD	
H.T (2)	0.52 ± 0.11	0.52 ± 0.11	0.942
H.T (5)	0.45 ± 0.11	0.46 ± 0.12	0.462
H.T (A)	0.60 ± 0.20	0.62 ± 0.23	0.613
CEJ—C	2.21 ± 0.77	2.24 ± 0.79	0.661
S.T (C)	0.75 ± 0.22	0.76 ± 0.20	0.531
S.T (2)	0.50 ± 0.12	0.50 ± 0.16	0.733
S.T (5)	0.53 ± 0.15	0.53 ± 0.16	0.573
F G—C	3.17 ± 0.82	3.19 ± 0.86	0.811
F G—CEJ	0.99 ± 0.58	1.01 ± 0.53	0.770

HT: hard buccal tissue, CEJ-C: distance from cementoenamel junction to alveolar crest, ST: soft buccal tissue, FG—C: distance from free gingiva to alveolar crest, FG—CEJ: distance from free gingiva to cementoenamel junction, and SD: standard deviation.

**Table 2:** Comparison of hard and soft tissue thicknesses in the maxillary anterior segment based on gender.

Quadrants	Female	Male	Significance level
	Mean ± SD	Mean ± SD	
H.T (2)	0.52 ± 0.11	52.0 ± 11.0	0.776
H.T (5)	0.42 ± 0.13	0.45 ± 0.09	^*∗*^0.035
H.T (A)	0.61 ± 0.23	0.62 ± 0.20	0.963
CEJ—C	2.18 ± 0.77	2.27 ± 0.80	0.623
S.T (C)	0.76 ± 0.21	0.75 ± 0.22	0.642
S.T (2)	0.51 ± 0.16	0.49 ± 0.12	0.196
S.T (5)	0.52 ± 0.16	0.54 ± 0.15	0.178
F G—C	3.18 ± 0.92	3.18 ± 0.75	0.834
F G—CEJ	1.04 ± 0.58	0.96 ± 0.53	0.720

HT: hard buccal tissue, CEJ-C: distance from cementoenamel junction to alveolar crest, ST: soft buccal tissue, FG—C: distance from free gingiva to alveolar crest, FG—CEJ: distance from free gingiva to cementoenamel junction, SD: standard deviation.

**Table 3:** Comparison of hard and soft tissue thicknesses in maxillary anterior segment based on age group.

Quadrants	≤30 years	30–40 years	>40 years	Significance level
	Mean ± SD	Mean ± SD	Mean ± SD	
H.T (2)	0.54 ± 0.11	0.51 ± 0.11	0.52 ± 0.11	0.084
H.T (5)	0.48 ± 0.12	0.45 ± 0.10	0.44 ± 0.13	^*∗*^0.010
H.T (A)	0.68 ± 25.0	0.61 ± 0.21	0.57 ± 0.19	^*∗*^<0.001
CEJ—C	2 ± 0.74	2.11 ± 0.70	2.48 ± 0.81	^*∗*^<0.001
S.T (C)	0.72 ± 0.2	0.75 ± 0.23	0.79 ± 0.21	^*∗*^0.021
S.T (2)	0.47 ± 0.12	0.48 ± 0.12	0.53 ± 0.17	^*∗*^<0.001
S.T (5)	0.51 ± 0.15	0.54 ± 0.15	0.53 ± 0.16	0.206
F G—C	3.03 ± 0.70	3.13 ± 0.82	3.32 ± 0.91	^*∗*^0.009
F G—CEJ	1.05 ± 0.55	1.11 ± 0.63	1.22 ± 0.46	^*∗*^<0.001

HT: hard buccal tissue, CEJ-C: distance from cementoenamel junction to alveolar crest, ST: soft buccal tissue, FG—C: distance from free gingiva to alveolar crest, FG—CEJ: distance from free gingiva to cementoenamel junction, and SD: standard deviation.

**Table 4:** Comparison of hard and soft tissue thickness at different levels between central and lateral incisors and canine.

Tooth	Central	Lateral	Canine	Significance level
	Mean ± SD	Mean ± SD	Mean ± SD	
H.T (2)	0.52 ± 0.11	0.54 ± 0.11	0.51 ± 0.11	0.076
H.T (5)	0.48 ± 0.12	0.45 ± 0.12	0.43 ± 0.11	^*∗*^0.002
H.T (A)	0.59 ± 0.19	0.64 ± 0.23	0.60 ± 0.23	0.278
CEJ—C	2.11 ± 0.76	2.22 ± 0.74	2.34 ± 0.83	^*∗*^0.007
S.T (C)	0.78 ± 0.23	0.75 ± 0.22	0.73 ± 0.20	0.151
S.T (2)	0.52 ± 0.14	0.49 ± 0.17	0.48 ± 0.11	^*∗*^0.020
S.T (5)	0.54 ± 0.14	0.56 ± 0.17	0.49 ± 0.15	^*∗*^0.001
F G—C	3.13 ± 0.83	3.21 ± 0.84	3.20 ± 0.85	0.479
F G—CEJ	1.05 ± 0.58	1.07 ± 0.58	0.89 ± 0.50	^*∗*^0.007

HT: hard buccal tissue, CEJ-C: distance from cementoenamel junction to alveolar crest, ST: soft buccal tissue, FG—C: distance from free gingiva to alveolar crest, FG—CEJ: distance from free gingiva to cementoenamel junction, and SD: standard deviation.

**Table 5:** Correlation of hard and soft tissue thickness in maxillary anterior segment based on tooth and quadrant.

Quadrants	Teeth	The degree of correlation at the 2 mm level from the crest (*P* value)	The degree of correlation at the 5 mm level from the crest (*P* value)
Right	Central	0.465 ^*∗*^(<0.001)	0.157 (0.165)
	Lateral	−0.02 (0.858)	0.156 (0.166)
	Canine	0.084 (0.460)	0.250 ^*∗*^(0.025)

Left	Central	0.272 ^*∗*^(0.015)	0.167 (0.140)
	Lateral	0.128 (0.258)	0.184 (0.102)
	Canine	−0.124 (0.272)	0.344 ^*∗*^(0.002)

HT: hard buccal tissue, CEJ-C: distance from cementoenamel junction to alveolar crest, ST: soft buccal tissue, FG—C: distance from free gingiva to alveolar crest, FG—CEJ: distance from free gingiva to cementoenamel junction, and SD: standard deviation.

## Data Availability

The data used to support the findings of this study are available from the corresponding author upon request.
